# Scopolaminebutyl given prophylactically for death rattle: study protocol of a randomized double-blind placebo-controlled trial in a frail patient population (the SILENCE study)

**DOI:** 10.1186/s12904-018-0359-4

**Published:** 2018-09-07

**Authors:** Harriëtte J. van Esch, Lia van Zuylen, Esther Oomen–de Hoop, Agnes van der Heide, Carin C. D. van der Rijt

**Affiliations:** 1 Erasmus MC Cancer Institute, Department of Medical Oncology, P.O. box 2040 3000CA, Rotterdam, the Netherlands; 2 Laurens Cadenza, Rotterdam, the Netherlands; 3 Erasmus MC, department of Public health, Rotterdam, the Netherlands; 4 Comprehensive Cancer organization, Rotterdam, the Netherlands

## Abstract

**Background:**

Death rattle (DR), caused by mucus in the respiratory tract, occurs in about half of patients who are in the dying phase. Relatives often experience DR as distressing. Anticholinergics are recommended to treat DR, although there is no evidence for the effect of these drugs. Anticholinergic drugs decrease the production of mucus but do not affect existing mucus. We therefore hypothesize that these drugs are more effective when given prophylactically.

**Methods:**

We set up a randomized double-blind, placebo-controlled, multi-center study evaluating the efficacy of prophylactically given subcutaneous scopolaminebutyl for the prevention of DR in the dying phase. The primary outcome is the occurrence of DR defined as grade ≥ 2 according to the scale of Back measured by a nurse at 2 consecutive time points with an interval of 4 h. Secondary outcomes include adverse effects, quality of dying, quality of life in the last three days and bereavement. A sub-study will explore the experience of participating in a clinical trial in the dying phase from the perspective of relatives. Four hospices will include 200 patients.

**Discussion:**

This is the first double-blind placebo-controlled study to prevent DR in patients in the hospice setting. Research in dying patients is challenging. We will apply ethical and organizational strategies as suggested in the literature.

**Trial registration:**

The trial is retrospectively registered in the Dutch Trial register, identifier NTR 6438 June 2017. EudractCT number 2016–002287-14.

## Background

Death rattle is defined as ‘noisy breathing caused by the presence of mucus in the upper respiratory tract’ [1, 2]. This phenomenon is a common symptom in the last days of life, where 12–92% of patients in the dying phase have been reported to develop death rattle [3]. In a recently completed study in our own hospital, death rattle was reported at least once in 40% (*n* = 149) of patients during the dying phase, and in 35% (*n* = 130) of patients during the last 24 h of life (Lokker ME, Heide Avd, Oldenmenger WH, Rijt CCDvd, Zuylen Cv: Hydration and symptoms in the last days of life, submitted). Nurses rated the symptom as moderately severe in 23% and as severe or very severe in 39% of these patients, according to the scale of Back [4].

Professionals assume that patients do not experience distress from death rattle because their consciousness is reduced in the dying phase [5]. Reduced consciousness is the main underlying cause of death rattle, as patients are no longer able to cough up mucus [5]. For the relatives, the rattling noise is unpleasant and disturbing, and they may fear that their loved one is suffering and might be choking [3, 6]. Sixty-six percent of the relatives of patients who died in a palliative care unit indicated to experience death rattle as “very stressful” and 53% of them said that there is a great need to improve the care for and treatment of death rattle [7].

Anticholinergic medication is considered the standard treatment for death rattle. Treatment of death rattle is regarded as an essential intervention in the care of dying patients by specialists in palliative care [8]. However, evidence for the efficacy of anticholinergics is scarce. Until now, two randomized placebo-controlled trials on anticholinergics for the treatment of death rattle have been published, both with important limitations. One study was clearly underpowered with the inclusion of seven patients [7], whereas the other study only used a single dose of the short-acting anticholinergic drug atropine [8]. Both trials found no differences in the intensity of death rattle and side effects between patients receiving anticholinergics and placebo. Another study compared the efficacy of three different anticholinergic drugs and found no differences between the drugs on the presence and intensity of death rattle [9]. The Dutch guideline on care for dying patients is cautious in advising the use of medication to treat death rattle [1]. Its recommended approach involves awareness, posture changes in bed and extensive pro-active explanation to and communication with relatives. Only if the rattle is still perceived as burdensome by relatives, anticholinergics may be considered.

Anticholinergics decrease the production of mucus but do not affect existing mucus [10]. These drugs may therefore be more effective if they are administered prophylactically [9–11], before death rattle occurs. [12] Scopolaminebutyl is the most frequently prescribed anticholinergic drug for death rattle in the Netherlands. We therefore designed a randomized controlled trial (RCT) to answer our research question: is scopolaminebutyl, when given prophylactically, effective in preventing death rattle? In this paper, we describe the protocol for this study and show how we addressed the medical-ethical rules and Good Clinical Practice guidelines [13] for this vulnerable patient population.

To perform an RCT in patients in the dying phase is a challenge. First, people in the last stage of their lives can be considered vulnerable and the ethics of asking vulnerable patients to participate in research is still a topic of debate [14–16]. Secondly, including sufficient numbers of patients has been demonstrated to be a challenge in palliative and end-of-life care research [17, 18]. And finally, a recent review showed that gatekeeping is a serious threat to performing research in palliative care [19]. We used several strategies that were suggested in the literature to address these problems.

## Methods

### Study design

The SILENCE study is a randomized double-blind, placebo-controlled, multi-center study to measure the efficacy of prophylactically administered scopolaminebutyl in the prevention of death rattle in the dying phase. This study will be conducted in four hospices. The trial is designed in accordance with the Good Clinical Practice guidelines and according to the general ethical principles outlined in the Declaration of Helsinki [13]. The trial is retrospectively registered in the Dutch Trial register, identifier NTR 6438; EudractCT number 2016–002287-14. The study incorporates a sub-study: a questionnaire will be sent to relatives to gain insight in how they experienced participation of their beloved one in research.

### Study population

Patients who are admitted to one of the participating hospice facilities will be asked for participation. Inclusion and exclusion criteria are described in Table 1. When the multidisciplinary team agrees that the dying phase has started, exclusion criteria will be checked again to see whether the patient is still eligible to participate.

**Table 1:** Inclusion/exclusion criteria

Inclusion criteria	Exclusion criteria
Patient is admitted to one of the participating hospices	Patient shows signs of active respiratory infection
Patient is aware that the admission will be up to death	Patient has a tracheostomy or tracheal cannula
Patient’s life expectancy at admission is at least 3 days	Patient uses systemic anticholinergic drug(s) or octreotide (at admission or when the dying phase starts)
Patient shows no signs of disturbed consciousness	Patient has death rattle grade 1 or higher on the scale of Back at the start of the dying phase.

### General procedure

#### Regular hospice care

In the Netherlands, patients who are admitted to a hospice facility in principle have a life expectancy of less than three months and stay there until death. Standard procedure at admission includes an interview about their symptoms, care needs and expectations, by both a doctor and a nurse.

Marking of the start of the dying phase, the last 2–5 days of life, is based on recognition of a number of signs by the nurses and doctors of the hospice: the patient is bedbound, only able to take sips of fluid, no longer able to swallow and take oral medication, and/or subcomatose.

When this state is recognized, the patient and his family are informed that death is imminent and they receive explanation about the care given in these last days and what symptoms might occur. They also receive a leaflet with written information. The recognition of the dying phase is a signal to start the Care Program for the Dying (CPD). The CPD is a digital, structured template for care in the last days and hours of life. This template defines goals of care in all four domains of palliative care (e.g. on pain, dyspnea, restlessness, nausea, death rattle, oral hygiene, needs of family on psychological/spiritual domain, needs of the patients on psychological/spiritual domain). The nursing staff evaluates these aspects of care and symptoms every four hours. They report their observations in the CPD. The observations are continued until the patient dies. The CPD is standard of care in the hospice facilities that participate in this study.

#### Study

For this study, standard care procedures in the hospice facilities are extended with some extra steps:

1. At admission: the doctor assesses if the patient meets the inclusion criteria of the study. If so, the patient is informed about the study and invited to think about participation. Written information will be given. After a period for reflection (maximum 48 h), the patient will get the opportunity for asking additional questions and, if he/she is willing to participate, to sign the informed consent form. The patient and family are aware of the fact that the study will not start immediately, but at the moment the dying phase is recognized. The doctor also informs the patient and relatives about the questionnaire which will be sent to relatives three months after the death of the patient.
2. Start of the dying phase: the actual study (the intervention) starts with the recognition of the dying phase. The exclusion criteria will be checked again. In case of eligibility, the first dose of the study medication scopolaminebutyl 20 mg (=1 ml) or placebo (=1 ml NaCl 0.9%) will be administered subcutaneously. The study medication will be provided four times a day. The study medication is packed in numbered boxes.
3. Care program: the CPD is extended with extra observations related to the study. The extra observations include restlessness, scored according to the Vancouver Interaction and Calmness Scale (VICS score) [20] and death rattle, scored according to Back [4]. Nurses also report on the oral care given.
4. Relatives: three months after a patient’s death, relatives will be sent a questionnaire.

#### Integration of study procedures into regular care

The study procedures have been discussed with the medical and nursing staff of the participating hospices to assure integration of the study logistics in regular care procedures. These procedures are largely similar for the different hospices, but differ regarding the admission process, contacts with pharmacists and procedures after death. Standard care is given according to the guidelines of the Netherlands Comprehensive Cancer Organization. Only few adjustments were needed in the procedures around the recognition of the dying phase and the care given in the last days of life (e.g. oral care, posture changes when (death) rattle starts, symptom management). Staff of the hospices have much experience in recognizing the dying phase and in working with the CPD. All personnel has been involved in previous clinical studies.

#### Training hospice staff

The multidisciplinary teams received a training about GCP. Further, they were trained on site in implementing the study protocol, procedures and data management. The primary researcher (HvE) provided this training on site.

#### Substudy

Relatives will be sent a questionnaire three months after the death of the patient. This questionnaire consists of several general questions (demographic and non-demographic), questions about the patient’s quality of life and quality of death, the Dutch version of the inventory of traumatic grief (ITG) [21] and questions about how participation of the patient in the study affected the quality of care according to the relative. Relatives will also be invited to take part in in-depth interviews about their experience of participating in research in the dying phase.

### Informed consent procedure

When a patient meets the inclusion criteria, he/she will be informed about the study and asked to participate. A patient information leaflet (PIL) is provided and time is given to consider participation. After 48 h, the patient can ask further questions and is asked for consent. When the patient agrees, the PIL is signed twice, with one copy for the patient and one for the researcher. Patients are free to withdraw from the study at any moment after signing informed consent.

### Intervention

When the start of the dying phase is recognized, study medication (scopolaminebutyl 20 mg (=1 ml) or placebo (=1 ml NaCl 0.9%)) will be given four times a day subcutaneously. Treatment will be continued until death or until the occurrence of death rattle with a severity of grade ≥ 2 according to the scale of Back [4] at two consecutive measurements with an interval of 4 h. In the latter case, medication will be regarded as “not effective” and the study medication will be withdrawn. The patient will then further receive care as usual.

### Randomization

Block randomization with varying block sizes of 2–4 is applied to create a randomization list for the study medication (verum or placebo) within each center (i.e. randomization is stratified by center).

As a result, verum and placebo are equally distributed over both patients and sites.

### Outcome measures

#### Primary outcome

The percentage of patients who develop death rattle, defined as the occurrence of death rattle with a severity of grade ≥ 2 according to the scale of Back at two consecutive measurements with an interval of 4 h.

#### Secondary outcomes

- Time from the recognition of the dying phase until the occurrence of death rattle (in hours)
- Occurrence of adverse events, i.e. urinary retention, dry mouth and restlessness
- Quality of life during the last three days of life and quality of dying, both according to the attending nurse and relatives
- Bereavement of relatives
- Experience of relatives concerning the patient’s participation in a (double-blind, placebo-controlled) clinical trial

### Measurement instruments

Table 2 provides an overview of measurement tools used in the SILENCE study.

**Table 2:** Measurement tools

	Measurement tool
Death rattle	Scale of Back: 0 = no rattle, 1 = audible close to the patient, 2 = audible standing at the end of the bed, 3 = audible standing in the door opening [4]
Restlessness	Vancouver Interaction and Calmness Scale (VICS score): 5 questions about restlessness scored on a 6-point Likert scale [20]
Dry mouth/urinary retention	Reported observations in CPD (agreed standards, implemented for this study)
Grief	Inventory of Traumatic Grief: 30 questions about grief [21]
Quality of life	Numeric rating scale (0 = worst quality – 10 = best quality) [22]
Quality of dying	Numeric rating scale: 0 = worst quality – 10 = best quality); 15 qualitative descriptions (peaceful, quiet, shocking, painful, etc.) [22] to tick.

### Data collection

Data collection starts when the dying phase starts and the CPD is implemented. Collection of patient-related information will be continued until death. All data will be collected on electronic case report forms using ALEA™, an online electronic database application as implemented by the Clinical Trial Center of the Erasmus MC Cancer institute (CTC).

### Sample size calculation

For the power calculation we used data from a previous study. In a sample of 400 deaths, 39% of the patients developed a death rattle with a severity of grade ≥ 2 according to the scale of Back (personal communication). We aim to reduce the occurrence of death rattle from 39 to 19%. Given a two-sided significance level of 0.05 and 80% power, a total of 180 patients are required based on a continuity-corrected Chi-square test. Taking into account that 10% of the patients who have given informed consent are expected to drop out before the actual start of the study because of sudden death or the occurrence of exclusion criteria, 200 patients are needed in total. Turnover in the four hospice settings is high: in the first three months of 2015, 120 patients were admitted, that is about 480 per year. Based on a 40% participation rate (= 192 out of 480), recognition of the dying phase and application of the CPD in 80% of all consenting patients(= 153 of 192) and a drop out of 10% it is possible to include approximately 140 patients in a year. This means that an inclusion period of approximately 18 months is needed.

### Statistical analysis

All analyses will be performed in the most recent version of IBM SSPS Statistics or Stata. Analyses will follow the intention-to-treat principle. However, patients initially randomized but considered ineligible afterwards based on information that should have been available before randomization, will be excluded from all analyses. A *p*-value < 0.05 is considered to indicate statistical significance.

#### Primary study parameter analysis

The occurrence of death rattle with a severity of grade ≥ 2 according to the scale of Back in both groups will be compared using the Chi squared test or, if necessary (i.e., if an observed number ≤ 10 or an expected number < 5 occurs in one of the cells of the 2 × 2 table), the Fisher’s exact test, to test the statistical significance of differences.

#### Secondary study parameters analysis

The time until the occurrence of death rattle will be compared by means of the Kaplan-Meier method and the log-rank test. The occurrence of adverse events, i.e. urinary retention and dry mouth (yes/no) will be described and compared using the Chi squared test or, if necessary, Fisher’s exact test to assess statistical significance. Restlessness will be compared using the t-test or, if necessary, the Mann-Whitney U-test.

Quality of life during the last three days of life and quality of dying will be described and compared by means of a t-test (or Mann-Whitney U test if necessary). The qualitative descriptions of the quality of dying will be summarized. Bereavement of relatives according to the ITG and experience of relatives on the patient’s participating in a (double-blind, placebo-controlled) clinical trial will be analyzed with descriptive statistics, and compared using the Chi squared test for trends.

### Ethical approval

This study is approved by the Medical Ethics committee of the Erasmus MC Rotterdam, the Netherlands.

## Discussion

The SILENCE study is a double-blind placebo-controlled study on the effect of prophylactically administered scopolaminebutyl on the occurrence of death rattle. Performing such a randomized clinical study in the dying phase is challenging because of practical and ethical concerns. This paper addresses how we designed the clinical trial and set up the procedures to make the study feasible.

An RCT is the gold standard for demonstrating efficacy of medication. There is a need for RCTs investigating symptom relief in the dying phase. The SILENCE study will provide evidence on the treatment of death rattle and its effect on quality of dying [3, 6, 7].

We are aware of the importance of a worthy dying process. Dying is a unique and irreversible experience, that cannot be redone and has to proceed as optimal as possible, not only for the patient but also for their relatives. The way a loved one dies can leave deep marks in the memories of the relatives. In designing a prospective study for a common symptom such as death rattle we had to take into account several challenges.

The first issue concerns “gatekeeping”, related to the vulnerability of the patients. “Gatekeeping” prevents eligible patients to participate in research studies [18]. Kars found five groups of “gate-keepers”: care professionals, research ethics committees, health care facility managers, relatives and researchers. To avoid gatekeeping in our study, managers, care professionals and researchers working in the four hospices have been involved in the project, from formulating a research question which was clinically and practically recognizable to the nursing staff, in writing and commenting on the grant proposal, to rewriting the protocol to standard operating procedures. Above all efforts were made to arouse enthusiasm for the research question in the multidisciplinary teams.

In line with this, we appointed a local researcher from the local professional care teams. Although it is common in clinical trials in hospitals that health care professionals (e.g. medical doctors and nurse practitioners) are involved in research and act as principal investigators, hospice staff is not accustomed to performing research, which involves a risk of fall-out, disinterest, mistakes in the study procedures and gate-keeping. In the literature on research in palliative care, several authors recommend combining the task of investigator and medical doctor; moreover, they plead for integration of research with practical (end-of-life) care. Kaasa has pleaded for “combined efforts between specialists in palliative care and researchers in the basic science”, [18] whereas Prince Paul and Casarett have advised to integrate the tasks of research and medical care [16, 22]. This would lead to a better understanding of the research process in professional care teams and make performing research in the hospice setting more standard. We followed their advice and trained a medical doctor from each hospice as the local investigator, as is also prescribed by GCP guidelines. Further, the local medical doctors and nursing teams were involved in setting up the study protocol. Because we also discussed and prepared the procedures locally, the identity and working procedures of the hospices could be preserved. In addition, all administrative activities for this study have been taken into consideration. The workload does not significantly increase because we will use standard data collection instrument that is also used in regular care by the participating teams: the Care Program for the dying (CPD). Assessment of the occurrence of death rattle is fully integrated in this program.

The second issue concerns the consent procedure. We ask patients’ consent to participate in the study at their admission in the hospice, when they are still capable of understanding the information and able to decide whether they want to participate. They may withdraw at any moment after signing the informed consent form. The actual study will start at the recognition of the dying phase, at which moment most patients are not able of giving consent due to their deteriorating condition. Rees and colleagues stated that asking advanced consent seems to be an acceptable alternative procedure, allowing research in patients who are unable to give consent at the time of randomization [23]. Patients who are admitted to a hospice facility in the Netherlands typically stay there until death, which makes a strategy of advanced consent feasible.

Our third strategy involves evaluation of the views of relatives about participation of their loved one in research in the last days of life. We developed a questionnaire which will be sent to relatives three months after the death of the patient. The challenge will be to sensitively ask relatives for consent to send this questionnaire at the moment the patient is admitted to the hospice.

This study has a few limitations. One limitation is the recognition of the dying phase. Although the staff is very experienced, inter-individual variability can occur. In the training of the teams, there has been thorough attention for signals that can indicate that the dying process has started. Another limitation is that although all the strategies presented aim to ensure adequate inclusion rates, the turnover of patients in the hospices fluctuate over time.

## Conclusion

The SILENCE study will evaluate the efficacy of prophylactically given scopolaminebutyl for the prevention of death rattle in the dying phase. This may improve treatment and care at the end of life. Legal and medical-ethical requirements for clinical trials are extensive and rigorous. We have implemented strategies to comply with these requirements based on advice from the literature. The SILENCE study started recruitment in April 2017 and is expected to be completed within 2 years.

## Abbreviations

CPD: Care Pathway for the dying
CTC: Clinical Trial Centre
DR: death rattle
ITG: Dutch version of the inventory of traumatic grief
PIL: patient information leaflet
VICS: Vancouver Interaction and Calmness Scale
